# Interferon-gamma coordinates CCL3-mediated neutrophil recruitment *in vivo*

**DOI:** 10.1186/1471-2172-10-14

**Published:** 2009-03-19

**Authors:** Cynthia A Bonville, Caroline M Percopo, Kimberly D Dyer, Jiliang Gao, Calman Prussin, Barbara Foster, Helene F Rosenberg, Joseph B Domachowske

**Affiliations:** 1 SUNY Upstate Medical University, Syracuse, New York 13210, USA; 2 Laboratory of Allergic Diseases, National Institute of Allergy and Infectious Diseases, National Institutes of Health, Bethesda, Maryland 20892, USA; 3 Laboratory of Molecular Immunology, National Institute of Allergy and Infectious Diseases, National Institutes of Health, Bethesda, Maryland 20892, USA; 4 Proteus Technologies, 133 National Business Parkway, Suite 150, Annapolis Junction, Maryland, 20701, USA

## Abstract

**Background:**

We have shown previously that acute infection with the respiratory pathogen, pneumonia virus of mice (PVM), results in local production of the proinflammatory chemokine, CCL3, and that neutrophil recruitment in response to PVM infection is reduced dramatically in CCL3 -/- mice.

**Results:**

In this work, we demonstrate that CCL3-mediated neutrophil recruitment is coordinated by interferon-gamma (IFNγ). Neutrophil recruitment in response to PVM infection was diminished five-fold in IFNγ receptor gene-deleted mice, although neutrophils from IFNγR -/- mice expressed transcripts for the CCL3 receptor, CCR1 and responded functionally to CCL3 *ex vivo*. Similarly, in the absence of PVM infection, CCL3 overexpression alone could not elicit neutrophil recruitment in the absence of IFNγ. Interestingly, although supplemental IFNγ restored neutrophil recruitment and resulted in a sustained weight loss among CCL3-overexpressing IFNγ -/- mice, CCL3-mediated neutrophil recruitment alone did not result in the pulmonary edema or respiratory failure characteristic of severe viral infection, suggesting that CCL3 and IFN-γ together are sufficient to promote neutrophil recruitment but not pathologic activation.

**Conclusion:**

Our findings reveal a heretofore unrecognized hierarchical interaction between the IFNγ and CCL3, which demonstrate that IFNγ is crucial for CCL3-mediated neutrophil recruitment *in vivo*.

## Background

Most respiratory virus infections are relatively benign and self-limited events. However, infection with highly pathogenic viruses can result in more severe sequelae, in which disease progresses to respiratory failure due to uncontrolled inflammation, pulmonary edema, and damage to lung tissue [1-5].

As part of an ongoing effort to understand inflammatory responses during severe respiratory virus infection, we have developed an inhalation model using the natural rodent pathogen, pneumonia virus of mice (PVM). Originally identified by Horsfall and colleagues [6,7], PVM is a pneumovirus (family *Paramyxoviridae*) that is closely related to respiratory syncytial virus (RSV), and is among the few characterized mouse models of virus-induced acute respiratory distress syndrome (ARDS) [7-9]. Among the prominent features of this infection, a minimal intranasal inoculum (30 – 100 pfu) results in robust virus replication within bronchial epithelial cells that is accompanied by profound granulocyte recruitment. In the absence of pharmacologic intervention, PVM infection progresses to pulmonary edema and respiratory compromise, similar to the more severe forms of RSV infection experienced by human infants [10,11]. In our earlier studies, we identified the chemokine CCL3 (MIP-1α) as a crucial component of this inflammatory response. PVM not only elicits production of CCL3 by infected bronchial epithelial cells [12], mice devoid of CCL3 or its receptor, CCR1, recruit dramatically fewer neutrophils to airways [13]. Blockade of the CCL3/CCR1 proinflammatory signaling pathway in conjunction with antiviral therapy resulted in improved survival in response to an otherwise lethal virus inoculum [14,15]. As CCL3 is only one of several major pro-inflammatory signaling pathways activated by PVM infection [12], there is certainly the possibility of additive, synergistic, or hierarchical means to promote and to amplify the ongoing inflammatory response.

Although first identified as a component of the antiviral response to Sindbis virus [16], the role of the Th1 cytokine, interferon-γ (IFNγ) in pneumovirus infection remains uncertain. IFNγ is readily detected in bronchoalveolar lavage fluid and nasal washings from RSV-infected infants [17,18], and minimal or absent response has been correlated with poor clinical outcome [19-24]. IFNγ is also detected in BAL fluid of BALB/c mice in response to challenge with RSV virions [25,26] and plays a role in limiting the inflammatory response to secondary challenge and in generating the allergic histopathology in response to formalin-fixed RSV vaccine antigens and virion components [27,28]. Likewise, local production of IFNγ is a prominent response to PVM infection [12,29,30], although its role in modulating the primary inflammatory response has not yet been fully explored.

In this manuscript, we explore the role of IFNγ in modulating the inflammatory response to PVM infection, and utilize overexpression analysis to begin a dissection of the independent and interdependent contributions of both IFN-γ and CCL3 to the process of neutrophil recruitment *in vivo*.

## Results

### Microarray profiling of IFNγ expression in response to PVM infection

**Figure 1:**
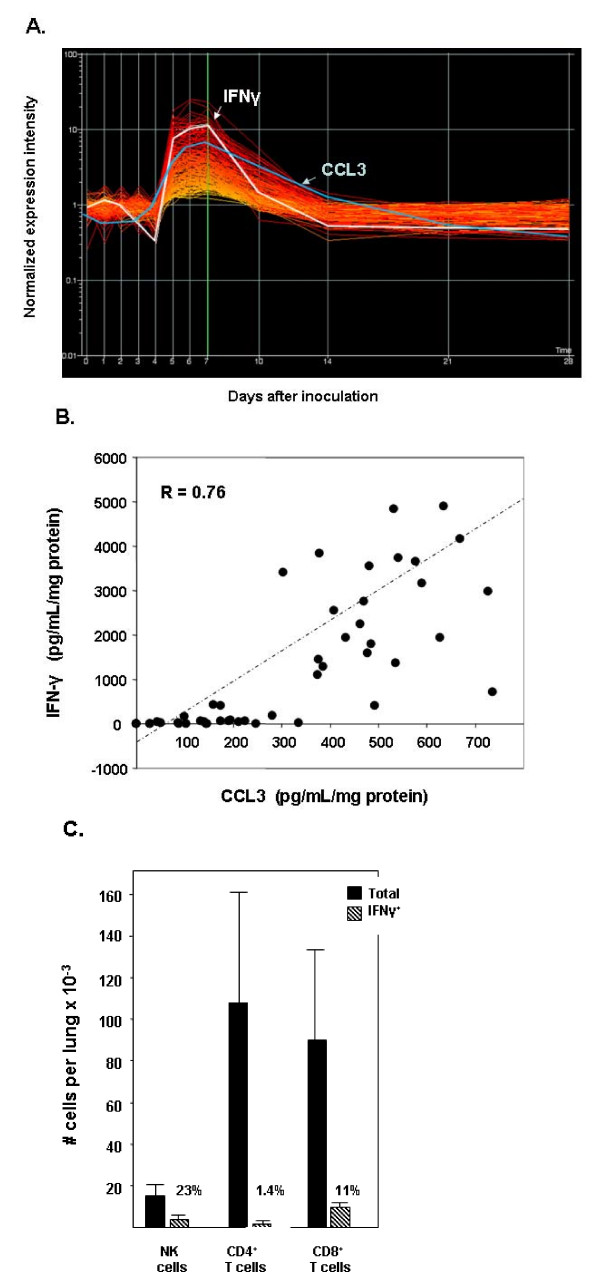
**(A) Expression of transcripts in mouse lung tissue in response to PVM infection: IFN-γ and IFN-γ correlating profiles**. Baseline expression in uninfected mice (day 0) is set at 1.0 and normalized expression (per gene, per chip) is shown for days 1 – 7, 10, 14, 21 and 28 after inoculation. Profiles of 203 transcripts with patterns that correlate with that the profile of IFN-γ (0.900 to 0.969) are shown in yellow to red, respectively, and identified by name in Table 1. The expression profile of CCL3 (MIP-1α), a chemokine crucial for neutrophil recruitment in response to PVM infection, is overdrawn with a blue line (correlation 0.965). **(B) Correlation of IFN-γ and CCL3 protein levels in individual PVM-infected mice**. IFN-γ and CCL3 detected by ELISA in lung tissue homogenates from individual mice days 0 – 28 after inoculation with 30 pfu PVM (n = 43) are as shown. **(C) IFNγ^+ ^NK and T cells detected in lungs of PVM-infected mice**. Total and IFNγ^+ ^NK cells, CD4^+ ^T cells, and CD8^+ ^T cells (± sd) detected per lung on day 6 after inoculation with 10 pfu PVM.

Transcript encoding the cytokine IFNγ was detected in mouse lung tissue at various time points in response to PVM infection [12]. In response to a non-lethal inoculum of PVM strain J3666, IFNγ mRNA was detected above baseline levels beginning on day 5. IFN-γ mRNA levels peak at day 7 after inoculation, and fall rapidly to baseline between days 7 – 14. Shown in Figure 1A are profiles of the 203 transcripts (of total 45,101 transcripts on the 430_2 mouse chip) that display kinetic expression correlations of 0.900 or greater with the IFN-γ profile, as per the 'find similar' algorithm of Genespring GX 7.3. Selected transcripts, categorized by function, are listed in Table 1. Among the transcripts that correlate with the IFNγ profile are 17 characterized interferon-response genes. Most intriguing is the close correlation (0.965) between the expression patterns of IFNγ and CCL3 (MIP-1α). CCL3 is essential for granulocyte recruitment in response to PVM infection [13]. As shown in Figure 1B, there is a significant correlation between levels of immunoreactive IFNγ and CCL3 in lung tissue from individual PVM-infected mice.

**Table 1:** Expression profiles that correlate with IFN-γ in PVM-infected mouse lung tissue.

**Transcript**	**Symbol**	**Acc. No.**	**Correl.**
**Interferon-γ and related transcripts**			
Interferon-γ	Ifng	K00083	1.000
Interferon inducible protein 1	Ifi1	NM_008326	0.961
Interferon-stimulated protein	Isg20	BC022751	0.953
Interferon-γ induced GTPase	Igtp	NM_018738	0.953
Interferon-induced transmembrane protein 6	Ifitm6	BB193024	0.950
CXC chemokine ligand 11 (IP-9)	Cxcl11	NM_019494	0.949
Interferon inducible protein 47	Ifi47	NM_008330	0.940
Interferon activatible protein 203	Ifi203	AI607873	0.932
Interferon activated gene 205	Ifi205	AI481797	0.929
Interferon induced protein with tetratricopeptide repeats 1	Ifit1	NM_008331	0.929
Interferon consensus sequence binding protein 1	Icsbp1	BG069095	0.926
Interferon regulatory factor 7	Irf7	NM_016850	0.922
Interferon activated gene 205	Ifi205	AI481797	0.916
Interferon regulatory factor 5	Irf5	NM_012057	0.914
Interferon activated gene 203	Ifi203	NM_008328	0.910
Interferon-induced protein with tetratricopeptide repeats, 3	Ifit3	NM_010501	0.909
Interferon induced protein with tetratricopeptide repeats 2	Ifit2	NM_008332	0.901

**Other inflammation-associated transcripts**			

CC Chemokine ligand 3 (MIP-1α)	Ccl3	NM_011337	0.965
Toll-like receptor 2	Tlr2	NM_011905	0.959
Interleukin-13 receptor alpha 1	Il13ra1	S80963	0.959
Suppressor of cytokine signaling 3	Socs3	NM_007707	0.951
Galectin-9	Lgals9	NM_010708	0.948
Interleukin-1 receptor antagonist	Il1rn	M57525	0.947
Regulator of G-protein signaling 19 interacting protein 1	Rgs19ip1	NM_018771	0.943
Interleukin-6	Il6	NM_031168	0.937
CD244 natural killer cell receptor 2B4	Cd244	NM_018729	0.928
CSF2 receptor	Csf2rb2	NM_007781	0.928
Fc receptor, IgG, high affinity, I	Fcgr1	AF143181	0.926
CC chemokine receptor 1	Ccr1	AV231648	0.926
Pentaxin-related gene	Ptx3	NM_008987	0.926
CXC chemokine ligand 13 (BLC)	Cxcl13	AF030636	0.921
CXC chemokine ligand 2 (MIP-2α)	Cxcl2	NM_009140	0.919
CXC chemokine ligand 5 (ENA-78)	Cxcl1	BB554288	0.914
Arginase II	Arg2	NM_009705	0.904
Signal transducer and activator of transcription 1	Stat1	AW214029	0.904
Regulator of G-protein signaling 1	Rgs1	NM_015811	0.903
CC chemokine receptor-like 2	Ccrl2	AJ318863	0.902

**Various**			

Membrane-spanning 4-domains, subfamily A, member 8A	Ms4a8a	NM_022430	0.969
Chondroitin sulfate proteoglycan 2	Cspg2	BM251152	0.963
Fas death domain-associated protein	Daxx	NM_007829	0.960
O-acyltransferase domain containing 1	Oact1	AV366860	0.960
Mitogen activated protein kinase kinase kinase kinase 1	Map4k1	BB546619	0.960
Lymphocyte cytosolic protein 2	Lcp2	BC006948	0.959
Solute carrier family 15, member 3	Slc15a3	NM_023044	0.956
Indoleamine-pyrrole 2,3 dioxygenase	Indo	NM_008324	0.954
Proteosome subunit beta type 9	Tap1	AW048052	0.952
Phospholipase A1 member A	Pla1a	NM_134102	0.949
Methylene tetrahydrofolate dehydrogenase	Mthfd2	BG076333	0.949
Pre-B colony enhancing factor 1	Pbef1	AW989410	0.948
Thioredoxin reductase 1	Txnrd1	BB284199	0.948
CGG triplet repeat binding protein 1	Cggbp1	BI080272	0.945
Sphingosine kinase 1	Sphk1	AF068749	0.944
Pyrophosphatase	Pyp	NM_026438	0.944
2'-5' oligoadenylate synthetase 1G	Oas1g	BC018470	0.943
Ubiquitin D	Ubd	NM_023137	0.943
Apolipoprotein D	Apod	NM_007470	0.940
Membrane-spanning 4-domains, subfamily A, member 4C	Ms4a4b	NM_029499	0.936
AT rich interacting domain 5A	Arid5a	BC027152	0.935
Hemopoietic cell kinase	Hck	NM_010407	0.933
Histocompatibility 2, complement component factor B	H2-Bf	NM_008198	0.933
ATP binding cassette	Abcc5	BB436535	0.933
Cholesterol 25-hydroxylase	Ch25h	NM_009890	0.932
BING 4 protein	Bing4	C78559	0.932
Thymidylate kinase, LPS inducible	Tyki	AK004595	0.930
Tripartite motif protein 30	Trim30	BM240719	0.929
Tissue specific transplantation antigen 30	Tsta3	NM_031201	0.929
Syndecan binding protein	Sdcbp	AV227603	0.928
Prostaglandin-endoperoxide synthase 2	Ptgs2	M94967	0.926
Traf binding protein	T2bp	BB277065	0.925
Two pore segment channel 2	Tpcn2	BC025890	0.925
Early growth response 2	Egr2	X06746	0.925
GLI pathogenesis-related 2	Glipr2	BM208214	0.925
Cytochrome p450, family 7, subfamily b	Cyp7b1	NM_007825	0.924
Rab20, Ras oncogene	Rab20	BG066967	0.923
Solute carrier 39	Slc39a14	BB399837	0.922
Dual specificity phosphatase 3	Dusp3	BQ266434	0.922
Ribosome binding protein 1	Rrbp1	AF273691	0.922
Spermidine synthase	Srm	NM_009272	0.921
Ubiquitin-specific protease 18	Usp18	NM_011909	0.920
Lipocalin	Lcn2	X14607	0.920
Jun-B oncogene	Junb	NM_008416	0.919
Guanylate nucleotide binding protein 3	Gbp3	NM_018734	0.919
Pre-B cell colony-enhancing factor 1	Pbef1	AW989410	0.917
Membrane-spanning 4-domains subfamily A, member 6B	Ms4a6b	NM_027209	0.917
SLAM family member 7	Slamf7	AK016183	0.915
Ras and Rab interactor 1	Rin1	BC011277	0.915
Class II transactivator	C2ta	AF042158	0.913
Myxovirus resistance I	Mx1	M21039	0.910
Chloride channel calcium activated 2	Clca1	AF108501	0.910
Rap2C, member of RAS oncogene family	Rap2c	AK008416	0.910
Tumor necrosis factor, alpha induced protein 2	Tnfaip2	NM_009396	0.908
SLAM family member 8	Slamf8	BC024587	0.908

The microarray analysis software package, Genespring GX 7.3 'find similar' function was used to inspect all transcript profiles for patterns related to that displayed by IFN-γ. The minimum correlation considered to be similar was set at 0.900 (see Figure 1A).

### Detection IFNγ^+ ^NK and T cells in PVM infected lung tissue

Both total and IFNγ^+ ^subsets of NK cells, CD4^+ ^and CD8^+ ^T cells were enumerated in single cell suspensions of lung tissue from PVM-infected BALB/c mice evaluated at day 6 after inoculation with 10 pfu PVM strain J3666 [Figure 1C]. Only a small fraction (<2%) of the CD4^+ ^T cells detected at this time point stained positively for IFNγ, in contrast to the larger fraction of IFNγ^+^CD8^+ ^T cells detected (9.9 ± 0.6 × 10^3 ^cells, 11% of total CD8^+ ^T cells). Interestingly, 23% of the total NK cells (3.4 ± 0.9 × 10^3 ^cells) stained positively for IFNγ, an increase from 0.3 ± 0.08 × 10^3 ^cells, or 4% of the total NK cells detected in a single lung from uninfected mice (data not shown).

### IFNγ-dependent responses to PVM infection

**Figure 2:**
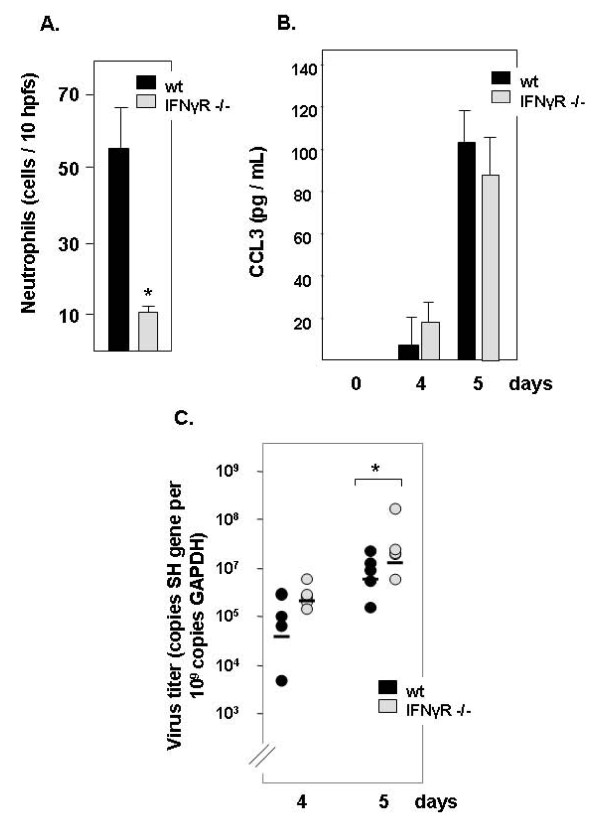
**Neutrophil recruitment in response to PVM infection is diminished in IFN-γR gene-deleted mice**. **(A) **Neutrophils detected in BAL fluid 5 days after inoculation; hpf, high power field; *p < 0.001;**(B) **Detection of CCL3 in BAL fluid; *p < 0.001 **(C) **Virus copy number detected in lung tissue determined by quantitative RT-PCR.

Wild type and IFNγ receptor gene deleted (IFNγR -/-) mice were infected with PVM and various parameters relating to the inflammatory response were assessed. Neutrophil recruitment to the airways was markedly diminished in IFNγR -/- mice [Figure 2A], reduced from 54 ± 11 per 10 hpfs among wild type to 10 ± 1.3 hpfs among IFNγR -/- mice, as determined on cytospin preparations of cells in BAL fluid (p < 0.001). These findings are consistent with those of Frey and colleagues [30], who described reduced inflammation in association with reduced IFNγ production in the lungs of PVM infected, T-cell deficient mice. Given our earlier studies on the essential role of CCL3 in eliciting neutrophil recruitment, it is interesting to note that the absence of IFNγ signaling had no impact on local production of this chemokine in response to PVM infection [Figure 2B]. IFNγ was also detected in response to PVM infection in both wild type and in IFNγR-/- mice, albeit at higher levels among the latter group, most likely due to the absence of feedback inhibition (data not shown). The diminished neutrophil recruitment, while significant, was not as profound as that observed in mice subjected to complete blockade of CCL3-mediated signaling, in which we observed 10^4^-10^5 ^fold-diminished neutrophil recruitment [14,15]. As might be anticipated from the diminished inflammatory response, we observe a statistically significant increase in virus titer among the IFNγR-/- mice [Figure 2C], although this difference is likewise not as dramatic as that observed in response to complete blockade of CCL3 signaling.

### Receptor expression and responses of neutrophils from IFNγR gene-deleted mice

**Figure 3:**
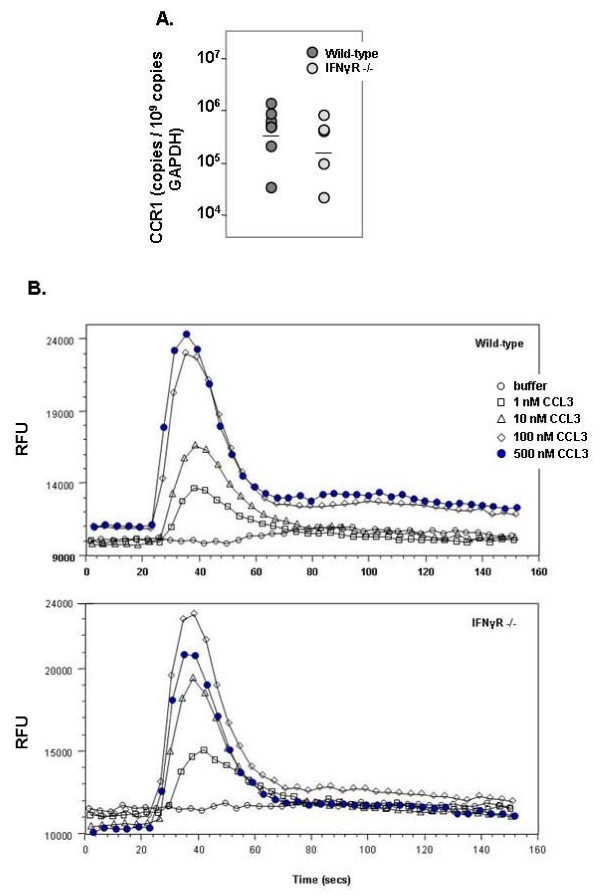
**Comparison of wild type and IFNγR gene-deleted neutrophils**. **(A) **Expression of CCR1 transcript in wild type and IFNγR gene-deleted neutrophils (n = 9 and 6 independent samples, respectively) determined by quantitative RT-PCR; horizontal line denotes mean copy number. **(B) **Calcium flux (RFU) measured in response to increasing concentrations (0 – 500 nM) of CCL3.

As part of an initial attempt to determine whether neutrophils from IFNγR -/- mice were capable of responding to CCL3, we explored receptor expression and ligand-mediated calcium flux in neutrophils isolated from both gene-deleted and wild type mice. As shown in Figure 3A, both wild type and IFNγR-/- neutrophils express transcripts encoding CCR1, the major receptor for CCL3; no significant difference in absolute copy number was determined. Likewise, CCL3 induced dose-dependent intracellular calcium flux in both gene-deleted and wild type neutrophils [Figure 3B], demonstrating that neutrophils from IFNγR-/- mice have the innate capacity to respond to this chemoattractant ligand; the EC50s and maximum calcium fluxes detected were indistinguishable between the wild type and gene-deleted strains.

### Overexpression of CCL3

**Figure 4:**
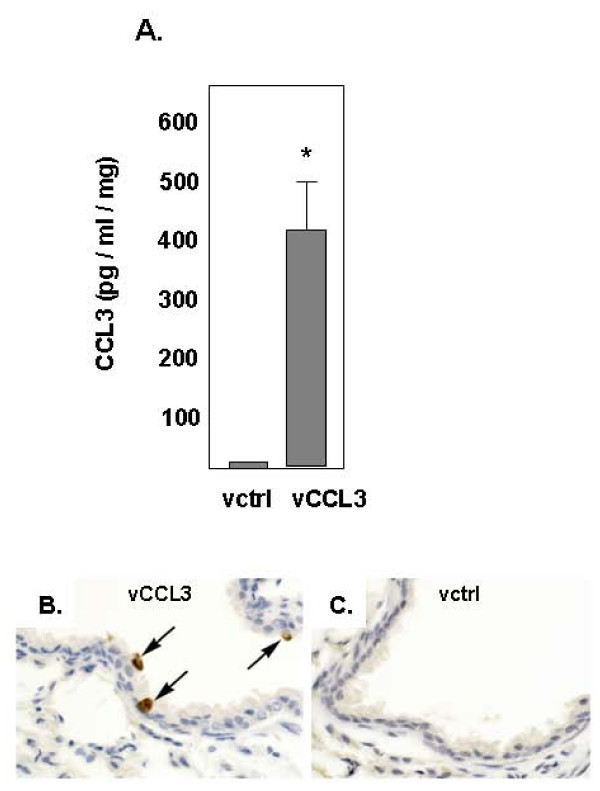
**Heterologous expression of CCL3 in mouse lungs**. **(A) **Detection of immunoreactive CCL3 in lungs of mice on day 9 after challenge via intranasal inoculation with the CCL3 overexpression vector (vCCL3) or control vector (vctrl), *p < 0.01. **(B) **Lung tissue from mice challenged with vCCL3, immunohistochemical localization of CCL3 within bronchiolar epithelial cells (at arrows), **(C) **Lung tissue from mice challenged with vctrl.

In order to examine the independent and interdependent contributions of CCL3 and IFNγ to the process of neutrophil recruitment *in vivo*, we generated a method for overexpression of CCL3 *in vivo*. CCL3 was detected in lung tissue homogenates [Figure 4A], reaching levels similar to those detected in lung tissue of mice in response to PVM infection [12]. Immunoreactive CCL3 was detected in bronchial epithelial cells [Figure 4B]. No CCL3-positive cells were detected in lung tissue from mice challenged with control vector (vctrl) [Figure 4C].

### Inflammatory responses to IFNγ and CCL3

**Figure 5:**
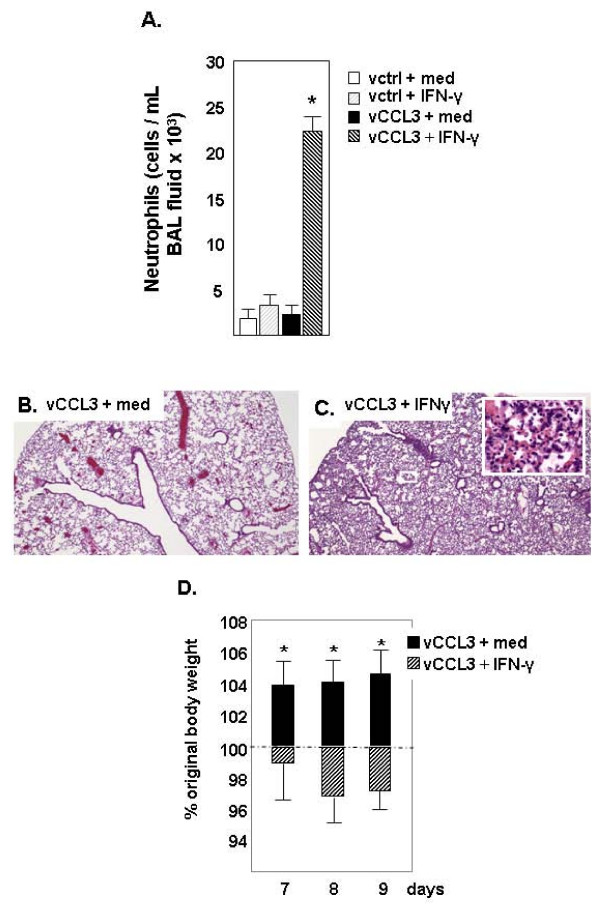
**Neutrophil recruitment in response to CCL3 is ablated in IFN-γ gene-deleted mice**. **(A) **Neutrophils detected in BAL fluid of IFNγ gene-deleted (IFNγ -/-) mice (+vctrl +med (medium; RPMI + 10% FCS vehicle control); open bar), + IFNγ (+vctrl + IFNγ, grey-shaded bar), +vCCL3 +med (black bar), or +vCCL3 + IFNγ (black-shaded bar); *p < 0.01 vs. other conditions, day 9 after challenge with vCCL3 or vctrl. **(B, C) **Microscopic images of lung tissue from IFN-γ -/- mice challenged with **(B) **vCCL3 + med or **(C) **vCCL3 + IFNγ; original magnification, 20×. Inset, original magnification 63×, documenting neutrophil recruitment. **(D) **Change in body weight in response to CCL3 overexpression ± IFN-γ; *p < 0.01 at time points shown.

We examined neutrophil recruitment in response to CCL3 overexpression in IFNγ gene-deleted mice (IFNγ -/-) with and without IFNγ supplementation. As shown in Figure 5A, few neutrophils are detected in BAL fluid at baseline (vctrl) and no recruitment over baseline is observed in response to IFNγ alone. Likewise, overexpression of CCL3 in the absence of IFNγ does not elicit neutrophil recruitment. Neutrophil recruitment (~10 – fold over baseline) was observed in response to CCL3 expression only in the presence of IFNγ. At the microscopic level, no inflammation was observed in lung tissue of IFNγ -/- mice in response to CCL3 overexpression alone [Figure 5B]. In contrast, significant pathology was observed in lung tissue of IFNγ -/- mice expressing CCL3 and supplemented with exogenous IFNγ. Findings include moderate peribronchiolar granulocytic infiltration and substantial parenchymal involvement but minimal edema fluid within the bronchioles and in the parenchymal tissue [Figure 5C]. Interestingly, weight loss is sustained among the mice overexpressing CCL3 while receiving supplemental IFNγ over the 9 day examination period [Figure 5D], but, despite the substantial inflammatory response, we observe no progression to respiratory failure up to and including t = 14 days.

## Discussion

In previous work, we demonstrated that the actions of the chemokine, CCL3, signaling via its receptor CCR1, were crucial for granulocyte recruitment to the lungs in response to PVM infection [13-15]; CCL3 has also been shown to be a crucial mediator of granulocyte recruitment in mouse models of influenza [31]. Paradoxically, CCL3 gene-deletion results in augmented neutrophil and eosinophil recruitment in response to *Cryptococcus neoformans *infection [32]. Here we show that CCL3-mediated neutrophil recruitment depends directly on IFNγ signaling, both in the setting of acute virus infection and in response to heterologous CCL3 expression in the respiratory epithelium.

Granulocyte recruitment is a primary finding in severe respiratory virus infection; activation of granulocytes can result in the release of proinflammatory cytokines and proteolytic enzymes that can contribute to the ongoing lung damage [33-37]. Interestingly, although neutrophils are recruited to the lung parenchyma in response to CCL3 via coordination by IFNγ, these cytokines alone clearly are not sufficient to induce the inflammatory state that ultimately promotes lung damage and respiratory failure. Thus, despite our findings demonstrating improved survival from PVM infection with CCR1 blockade [15], and those of He and colleagues [38], who likewise demonstrated that CCR1 antagonism provided protection against neutrophil-mediated lung injury in a mouse model of acute pancreatitis, the results presented here, in which we observe neutrophil recruitment but minimal clinical disease, suggest that neutrophil recruitment and neutrophil activation are to some extent distinct and discrete signaling events. It will be crucial to identify the proinflammatory mediators that activate and well as those that recruit neutrophils in order to have a complete picture of the proinflammatory state characteristic of PVM infection.

The experimental studies performed in this manuscript utilize both IFNγ and IFNγR gene-deleted mice, which are in BALB/c and C57BL/6 background strains, respectively. PVM infection has been explored systematically in several inbred strains of mice by Anh and colleagues [39] who determined that the C57BL/6 strain is somewhat more resistant to infection than BALB/c, but that both of these inbred strains can ultimately succumb to the sequelae of severe disease. We have used both of these strains extensively for our studies (reviewed in [7-9]) and both respond to PVM infection with robust virus replication in lung tissue, granulocyte recruitment and local production of proinflammatory cytokines, including CCL3 and IFNγ; no systematic differences, other than the aforementioned susceptibility to infection, have been detected.

Both CCL3 and IFNγ have been detected in human studies and in mouse models of other severe respiratory virus infections, including avian influenza, SARS coronavirus, and human respiratory syncytial virus [17,18,40-47], although the potential for interplay between these specific signaling pathways has not been considered previously. Our data suggest that that IFNγ and CCL3 signaling pathways, both crucial features of the response to pneumovirus infection, interact in a hierarchical fashion, as IFNγ does not elicit neutrophil recruitment on its own [Figure 5A], but is crucial for CCL3 to function effectively. Interactions between IFNγ and CCL3 may occur at the level of signal transduction, or via alterations to the neutrophil itself. As has been documented clearly, CCL3 can function alone to induce changes in calcium concentration and chemotactic responses in mouse neutrophils *in vitro *[48]. The current literature on interactions of IFNγ with granulocytes was recently reviewed [49]. Among the possibilities that may address our findings, Hansen and Finbloom [50] reported that human neutrophils express IFNγ receptors and Bonecchi and colleagues [51] have shown that human neutrophils respond to IFNγ with increased expression of a variety of mediators and receptors, including the primary CCL3 receptor, CCR1. It is unclear whether mouse neutrophils respond in a similar fashion, and whether or not these defined molecular responses take place *in vivo*, although we have shown here that neutrophils from IFNγR gene-deleted mice express transcripts for CCR1 and mobilize intracellular calcium in response to CCL3 when examined *ex vivo*. We have not yet explored the possibility that the IFNγ coordinates neutrophil recruitment in response to CCL3 in a more indirect fashion, possibly via one or more intermediary cytokines. An example of this phenomenon was reported by Khader and colleagues [52], who demonstrated that *Mycobacterium tuberculosis*-infected dendritic cells from IL-12p40 gene-deleted mice that were unresponsive to a CCL19 gradient were also overproducing the cytokine IL-10. Most intriguing, addition of IL-10 to wild-type dendritic cells reproduced the inhibited chemotaxis response.

## Conclusion

In summary, we demonstrate here that CCL3, a proinflammatory mediator produced in response to RSV and shown to be a crucial in recruiting neutrophils in response to the mouse pneumovirus, PVM, functions via a hierarchical relationship with IFNγ. Specifically, CCL3 recruits neutrophils to the lung *in vivo *only in coordination with IFNγ-mediated signaling pathways. The mechanism via which IFNγ modulates neutrophil responses to CCL3 is an intriguing subject for future exploration.

## Methods

### Microarray analysis

Generation of gene microrarray data was as described previously [12]. Data collected were evaluated using the microarray software analysis package Genespring GX 7.3. The 'find similar' function was used to inspect all 45,101 transcript profiles in order to detect kinetic profiles similar to that of IFNγ. The minimum correlation to be considered a similar profile was set at 0.900. The higher the correlation coefficient (maximum 1.000 for complete overlap), the more similar the gene expression profiles.

### Mouse, virus and vector stocks

BALB/c and C57BL/6 mice were purchased from Taconic Laboratories (Germantown, NY and Rockville, MD). Homozygous IFNγ gene-deleted (IFNγ -/-) mice [53] on a BALB/c background and IFNγ receptor gene-deleted (IFNγR -/-) mice [54] on a C57BL/6 background were purchased from Jackson Laboratories, Bar Harbor, ME. All animal studies were performed as per approved protocols CHUA #634 (SUNY Upstate) or LAD 8E (NIAID). PVM strain J3666 was passaged, stored and quantitated as described previously [13]. Mice were anaesthetized and inoculated by intranasal challenge with 30 – 100 plaque forming units (pfu) PVM also as previously described. For challenge with recombinant vectors (described as follows), dilutions of secondary stock aliquots of vCCL3 and vctrl (described in the section to follow) were prepared in RPMI cell culture medium. Under brief anaesthesia, mice were inoculated with 150 μl of stock (50 μl/dose × 3 doses) to achieve challenges of 1.0 – 1.5 × 10^11 ^pfu per mouse. On days indicated, mice in each challenge group were sacrificed by cervical dislocation and bronchoalveolar lavage (BAL) fluid, total lung protein and total lung RNA were harvested. For some experiments, mice received 15 μg recombinant murine IFNγ (R&D Systems, Minneapolis, MN) diluted in tissue culture medium (RPMI + 10% fetal calf serum) or tissue culture medium (vehicle) via intraperitoneal injection one day prior to intranasal challenge with the vCCL3 or vctrl which yielded 323 ± 28 pg IFNγ/mg lung on day 4 post-inoculation.

### Flow cytometric determination of IFNγ^+ ^NK and T cells in mouse lung tissue

Whole lungs of BALB/c mice (uninfected or day 6 after inoculation with 10 pfu PVM, n = 5 per datapoint) were cut into ~3 mm^3 ^pieces in HBSS buffer (Invitrogen) and pressed through a 100 micron cell strainer (BD Biosciences, San Jose, CA) to obtain single cell suspensions. Cells were suspended in RPMI-1640 medium supplemented with 10% fetal calf serum, 2 mM glutamine, 100 U/mL penicillin, 100 U/mL streptomycin, 50 μM 2-mercaptoethanol, 1 mM sodium pyruvate, and nonessential amino acids (all from Invitrogen) and incubated for 6 hrs at 37°C at a density of 1 × 10^6 ^cells/ml with 1 μM ionomycin, 20 ng/ml phorbol-12-myristate acetate (EMD Biosciences, San Diego, CA) and 10 μg/ml brefeldin A (Sigma-Aldrich Co., St. Louis, MO). DNAse I (Sigma) was added for 5 minutes and then cells were washed once and stained with violet LIVE/DEAD Fixable Dead Cell stain (Invitrogen) for 30 minutes on ice, washed in PBS, fixed in 4% PFA, and stored at -80°C until analysis. Intracellular cytokine staining was performed as described previously [55]. Cells were stained with I-Ad FITC, DX5-PE, CD3-PE-Cy5, CD4 PerCP/Cy5.5, IFNγ PE-Cy7, and CD8 APC-Cy7 (BD Biosciences) in PBS with 0.1% BSA, 0.1% saponin (Sigma) and 5% nonfat dry skim milk. Controls were stained with isotype matched antibodies. Samples were acquired with a 4-laser LSR II flow cytometer (BD Biosciences) and analyzed on FlowJo software (Tree Star, Inc., San Carlos, CA). Viable lymphocytes were identified by typical forward and side scatter and negative staining for LIVE/DEAD violet. T cells were identified as I-A^-^, CD3+ and either CD4^+ ^or CD8^+ ^as indicated; NK cells were identified as I-A^-^, CD3^-^, DX5+. Quadrant statistical markers were based on corresponding isotype matched controls. Samples consisted of a known fraction (typically 1/4) of the cells obtained from a whole lung, and the entire sample was analyzed (typically 2 – 4 × 10^5 ^events) yielding the absolute number of cells per lung.

### Isolation of neutrophils from wild type and IFNγR-/- mice

Neutrophils were isolated from wild type and IFNγR-/- mice as described [56]. Briefly, 2 mL intraperitoneal injection of thioglycollate was administered and 4 hours later, mice were sacrificed and cells were harvested by peritoneal flush with 10 mL PBS (without calcium or magnesium). Cells were washed, red blood cells lysed with distilled water, and viability determined at >95% by trypan blue exclusion. Further isolation via Ficoll/Hypaque density gradient centrifugation yielded neutrophil purities of 85 – 99% as determined by modified Giemsa staining of cytospin preparations.

### Absolute quantification of CCR1 expression

Total RNA was isolated from neutrophils elicited from wild type and IFNγR -/- mice using the RT^2 ^qPCR-Grade RNA Isolation Kit (SuperArray Bioscience Corporation). The cDNA was prepared using the 1^st ^strand cDNA Synthesis Kit for RT-PCR (AMV; Roche Applied Science). QPCR was performed using the TaqMan Universal PCR Maser Mix (Applied BioSystems) with primer-probe pairs for GAPDH (TaqMan Rodent GAPDH Control Reagents VIC probe Applied Biosysystems) or CCR1 (chemokine (C-C motif) receptor 1 Mm00438260_s1 FAM labled, Applied Biosystems). The standard curve for mouse GAPDH included serial dilutions of the DECA template GAPDH-Mouse probe (Ambion); the standard curve for mouse CCR1 included serial dilutions of the coding sequence (GenBank Accession # U28404) in pCEP4. Reactions were run in triplicate in the 7500 RealTime PCR System (Applied Biosystems); data presented as copies of CCR1 per 10^9 ^copies GAPDH.

### Intracellular calcium measurements

Intracellular calcium measurements were performed in a Benchtop Scanning Fluorometer and Integrated Fluid Transfer Workstation (Flexstation; Molecular Devices) as described [56]. Briefly, 2.5 × 10^5 ^thioglycollate-elicited neutrophils were suspended in 100 μl of Hank's buffered saline solution with 20 mM HEPES and 100 μl fluorescent dye (FLIPPER calcium 3 assay kit component A; Molecular Devices) in a 96 well plate. The cells were incubated at 37°C for 30 minutes, centrifuged for 5 minutes, and challenged with various concentrations of CCL3, buffer alone (negative control) or f-MLF (positive control). Changes in intracellular calcium concentration were recorded as relative fluorescence units (RFU).

### Construction vCCL3 and control (vctrl) overexpression vectors

Generation of overexpression vectors was accomplished using the commercially available AdEasy XL vector system (Stratgene, La Jolla CA) according to the manufacturer's instructions. Briefly, murine CCL3 (GenBank Accesion No. NM 011337) was ligated into the multiple cloning site of the shuttle vector, pShuttleCMV; the corresponding control plasmid, containing the β-galactosidase gene, was supplied with the kit. The constructs were linearized with *Pme *I and transformed into an *E. coli *strain, (BJ5183) which contains the replication-incompetent pAD-1 backbone. Transformants were selected for kanamycin resistance, and recombinants subsequently identified by restriction digestion. Once recombinants were identified, they were produced in bulk using the recombination-deficient bacterial strain, XL-10 Gold. Purified recombinant plasmid DNA was digested with *Pac *I to expose inverted terminal repeats and used to transfect AD-293 cells in which the deleted viral assembly genes are complemented *in vivo*. The resulting constructs, vctrl and vCCL3 were harvested from the transfected AD-293 cells when more than 90% of the monolayer exhibited cytopathic effects. Secondary stocks were produced in a similar fashion. Titration was performed by standard plaque assay. The concentration of secondary stocks reached titers of ~10^12 ^pfu/ml for each construct. Viral stocks were stored at -80°C prior to use.

### Bronchoalveolar lavage (BAL) and differential cell counts

At time points indicated, BAL fluids were harvested from 5 mice by trans-tracheal instillation and removal of pre-chilled phosphate-buffered saline with 0.25% bovine serum albumin (BSA; 0.80 ml instillation with recovery of 0.5 to 0.6 ml per mouse). Neutrophil counts were determined by visual inspection of methanol-fixed cytospin preparations stained with modified Giemsa (DiffQuik, Fisher Scientific, Pittsburgh PA).

### Chemokine and cytokine determinations

Concentrations of CCL3 and IFNγ were determined in BAL fluid (pg/ml) isolated as previously described [13] from five mice per datapoint, using commercially available ELISA kits (R&D Systems, Minneapolis, MN). Total protein was determined the Bradford colorimetric assay using bovine serum albumin standards

### Gross and microscopic pathology and immunohistochemical detection of CCL3

Paraffin blocks of formalin-fixed lung tissue from mice challenged with vctrl or vCCL3 were paraffin-embedded and sectioned. Standard hematoxylin and eosin staining of formalin-fixed tissue was performed by American Histolabs (Gaithersburg, MD). To detect CCL3 protein expression *in situ*, slides were incubated with a 1:50 dilution of goat anti-CCL3 (R&D Systems, Minneapolis, MN) followed by a 1:400 dilution of biotinylated rabbit anti-goat Ig and developing reagents (performed by Histoserv, Inc., Germantown, MD).

### Virus titer

Quantitative reverse transcriptase PCR to document PVM titer in mouse lung tissue was as described previously [57]. Datapoints are presented as copies of PVM SH gene per 10^9 ^copies GAPDH.

### Statistical analysis

Experimental datapoints were from triplicate samples, experiments replicated two to three times. Data were evaluated by Student's t-test or Mann-Whitney U-test as appropriate.

## Abbreviations

CCL3: CC chemokine ligand 3; IFNγ: interferon-gamma; IFNγR: interferon-gamma receptor; PVM: pneumonia virus of mice; RSV: respiratory syncytial virus; CCR1: CC chemokine receptor 1; BAL: bronchoalveolar lavage; βgal: beta galactosidase; NK: natural killer; CCL19: CC chemokine ligand 19; PFU: plaque forming units; RFU: relative fluorescence units.

## Authors' contributions

CB contributed to the initial design of the CCL3 overexpression studies and carried out the experimental work. CP contributed to the design of the virus infection studies and carried out the experimental work, also purified mouse neutrophils and determined CCR1 expression by quantitative RT-PCR. KD contributed to the design of the virus infection and neutrophil purification studies, reviewed the manuscript and assisted in design of the display items. JG assisted with the neutrophil purification protocol and performed the calcium transient studies. CP and BF designed and executed of the flow cytometry and intracellular cytokine staining studies. JD designed and provided direct overview of the CCL3 overexpression studies, and wrote the first draft of the manuscript. HR designed and provided direct overview of the virus infection studies, compiled the subsequent and final drafts of the manuscript and figures and oversaw the manuscript submission and revision.

All authors read and approved the final manuscript.

## Author Information

Joseph B. Domachowske, M. D. is Professor of Pediatrics, Microbiology and Immunology at SUNY Upstate Medical University, Syracuse, New York. Helene F. Rosenberg, M. D. is Senior Investigator and Section Chief in the Laboratory of Allergic Diseases, National Institute of Allergy and Infectious Diseases, National Institutes of Health, Bethesda, Maryland. Drs. Domachowske and Rosenberg have collaborated extensively, and together have developed the pneumonia virus of mice (PVM) model for the study of the sequelae severe respiratory virus infection in a rodent host.
